# Automated detection of myopic maculopathy from color fundus photographs using deep convolutional neural networks

**DOI:** 10.1186/s40662-022-00285-3

**Published:** 2022-04-01

**Authors:** Jun Li, Lilong Wang, Yan Gao, Qianqian Liang, Lingzhi Chen, Xiaolei Sun, Huaqiang Yang, Zhongfang Zhao, Lina Meng, Shuyue Xue, Qing Du, Zhichun Zhang, Chuanfeng Lv, Haifeng Xu, Zhen Guo, Guotong Xie, Lixin Xie

**Affiliations:** 1 Qingdao Eye Hospital of Shandong First Medical University, 5 Yanerdao Road, Qingdao, 266071 China; 2 State Key Laboratory Cultivation Base, Shandong Provincial Key Laboratory of Ophthalmology, Shandong Eye Institute, Shandong First Medical University & Shandong Academy of Medical Sciences, Qingdao, 266071 China; 3 Ping An Healthcare Technology, 9F Building B, PingAn IFC, No. 1-3 Xinyuan South Road, Beijing, 100027 China; 4 Shandong Eye Hospital of Shandong First Medical University, Jinan, 250021 China; 5 Rongcheng Eye Hospital, Weihai, 264200 China; 6 971 Hospital of PLA Navy, Qingdao, 266071 China; 7 Qilu Hospital of Shandong University (Qingdao), Qingdao, 266035 China; 8 Ping An Healthcare and Technology Company Limited, Shanghai, 200030 China; 9 Ping An International Smart City Technology Company Limited, Shenzhen, 518000 China

**Keywords:** Myopic maculopathy, Tessellated fundus, Pathologic myopia, Deep convolutional neural network, Color fundus image

## Abstract

**Background:**

Myopic maculopathy (MM) has become a major cause of visual impairment and blindness worldwide, especially in East Asian countries. Deep learning approaches such as deep convolutional neural networks (DCNN) have been successfully applied to identify some common retinal diseases and show great potential for the intelligent analysis of MM. This study aimed to build a reliable approach for automated detection of MM from retinal fundus images using DCNN models.

**Methods:**

A dual-stream DCNN (DCNN-DS) model that perceives features from both original images and corresponding processed images by color histogram distribution optimization method was designed for classification of no MM, tessellated fundus (TF), and pathologic myopia (PM). A total of 36,515 gradable images from four hospitals were used for DCNN model development, and 14,986 gradable images from the other two hospitals for external testing. We also compared the performance of the DCNN-DS model and four ophthalmologists on 3000 randomly sampled fundus images.

**Results:**

The DCNN-DS model achieved sensitivities of 93.3% and 91.0%, specificities of 99.6% and 98.7%, areas under the receiver operating characteristic curves (AUC) of 0.998 and 0.994 for detecting PM, whereas sensitivities of 98.8% and 92.8%, specificities of 95.6% and 94.1%, AUCs of 0.986 and 0.970 for detecting TF in two external testing datasets. In the sampled testing dataset, the sensitivities of four ophthalmologists ranged from 88.3% to 95.8% and 81.1% to 89.1%, and the specificities ranged from 95.9% to 99.2% and 77.8% to 97.3% for detecting PM and TF, respectively. Meanwhile, the DCNN-DS model achieved sensitivities of 90.8% and 97.9% and specificities of 99.1% and 94.0% for detecting PM and TF, respectively.

**Conclusions:**

The proposed DCNN-DS approach demonstrated reliable performance with high sensitivity, specificity, and AUC to classify different MM levels on fundus photographs sourced from clinics. It can help identify MM automatically among the large myopic groups and show great potential for real-life applications.

## Background

The prevalence of myopia is rising rapidly in many countries and poses a heavy public health burden and cost to society. By 2050, it is estimated that 50% and 10% of the world will have myopia and high myopia (HM), respectively [1, 2]. Pathologic myopia (PM) degenerates from HM and includes a set of principal alterations, such as excessive axial elongation of the globe, posterior staphyloma, optic disc changes, and myopic maculopathy (MM). These pathological changes, particularly MM, cause severe visual loss in patients with PM over time [3]. In fact, MM, also known as myopic macular degeneration, has become a major cause of visual impairment and blindness worldwide, especially in East Asian countries [4–9].

Early detection of MM, along with timely management and treatment, are essential for preventing vision loss [10]. However, there is a disproportionately low number of ophthalmologists available to manage high-risk groups, especially in underdeveloped countries [11, 12]. Automated detection of MM via artificial intelligence shows great potential to resolve this issue. In recent years, deep learning approaches such as deep convolutional neural networks (DCNN) have been successfully applied in the automated detection of common fundus diseases, such as diabetic retinopathy, age-related macular degeneration, glaucomatous optic neuropathy and retinopathy of prematurity, from color fundus photographs [13–20]. Inspired by this, DCNN models were applied to tackle the identification and classification of PM and the segmentation of PM-related lesions; feasibility was demonstrated using a limited dataset of 1200 color fundus images released by the International Symposium on Biomedical Imaging [21, 22]. Then, three medical studies that adopted deep learning methods to identify MM from fundus images were published in 2021 [23–25]. The sizes of datasets and the MM categories to be automatically distinguished varied in these studies. However, the reported performances showed visible declines in the external testing datasets in two studies [23, 24] and the number of fundus images used for external testing in another study [25] was also relatively small.

In this study we established a large database including over 50,000 color fundus images from a range of camera devices at multiple medical centers. A reliable DCNN approach was proposed to automatically identify MM with different severity levels and overcome the effects of variations in our large-scale database. We then conducted a series of comparison experiments to assess the performance of this approach.

## Patients and methods

This study was approved by the Institutional Review Board of Qingdao Eye Hospital of Shandong First Medical University and conducted in accordance with the Declaration of Helsinki. Informed consent was waived by the medical ethics committee due to the retrospective design with analysis of fully anonymous color fundus images.

## Data and grading

In this study, a total of 57,148 color fundus images from 29,230 patients were collected from ophthalmology clinics of six hospitals in China between May 2018 and May 2020. They were captured using different types of nonmydriatic fundus cameras (e.g., Topcon, Canon, Zeiss, Kowa, Syseye) that mainly adopted the 45° macula-centered imaging protocol. If there were two or more images taken for one unique eye, the latest image was reserved. Fundus images from four hospitals were used to develop the DCNN models, and those from the other two hospitals were reserved for external testing. Detailed characteristics of the datasets are summarized in Table 1.

**Table 1:** Dataset summary

Data source	Subject characteristics						Grading distribution			
	No. of images	No. of eyes	No. of individuals	Age Mean (SD)	Female no./total individuals (%)	Camera	No. of gradable images/total images (%)	No. of no MM/total gradable images (%)	No. of TF/total gradable images (%)	No. of PM/total gradable images (%)
Development dataset										
Qingdao Eye Hospital North Branch of Shandong First Medical University	15,857	15,857	8156	50.8 (11.3)	3809 (46.7)	Canon, Syseye	14,356 (90.5)	11,856 (82.6)	996 (6.9)	1504 (10.5)
Rongcheng Eye Hospital	15,683	15,683	7919	52.1 (15.4)	3857 (48.7)	Zeiss	13,876 (88.5)	10,647 (76.7)	1749 (12.6)	1480 (10.7)
Qilu Hospital of Shandong University (Qingdao)	4915	4915	2507	55.2 (13.2)	1243 (49.6)	Canon	4393 (89.4)	2987 (68.0)	620 (14.1)	786 (17.9)
No. 971 Hospital of the People’s Liberation Army	4139	4139	2184	59.8 (12.9)	1262 (57.8)	Topcon	3890 (94.0)	1896 (48.7)	902 (23.2)	1092 (28.1)
External testing datasets										
Shandong Eye Hospital of Shandong First Medical University	7869	7869	4024	54.5 (15.5)	1898 (47.2)	Topcon	7077 (89.9)	4884 (69.0)	1687 (23.8)	506 (7.2)
Qingdao Eye Hospital of Shandong First Medical University	8685	8685	4440	49.2 (17.5)	2264 (51.0)	Kowa	7909 (91.1)	6508 (82.3)	747 (9.4)	654 (8.3)

*MM* = myopic maculopathy; *TF* = tessellated fundus; *PM* = pathologic myopia; *SD* = standard deviation

According to the META-PM study [3], MM was divided into five categories according to the disease severity on color fundus images. They consist of “no myopic retinal lesions” (category 0), “tessellated fundus (TF) only” (category 1), “diffuse chorioretinal atrophy” (category 2), “patchy chorioretinal atrophy” (category 3), and “macular atrophy” (category 4). Several plus lesions supplement the META-PM categories, including lacquer cracks, myopic choroidal neovascularization, and Fuchs’ spot. In this study, no MM and TF were equivalent to category 0 and 1 respectively, while PM was defined as category 2 to 4 or presence of any plus lesions.

Images of poor quality (where ≥ 50% of the macular area was obscured caused by severe artifact, defocus blurring and too dark or too light illumination) or wrong field definition (not macula-centered fundus photographs) were excluded by means of manually reviewing fundus images. The remaining gradable images would be labeled as one of the three MM severity levels (no MM, TF, and PM). All fundus images in the development and external testing datasets were subjected to a multiple-tier labeling process performed by four reading ophthalmologists (LQQ, XSY, DQ, and ZZC) with over two years’ experience and two retinal experts (GY and LJ) with more than 15 years of clinical experience. Firstly, four reading ophthalmologists were informed of the grading criteria. They achieved a high level of agreement (κ ≥ 0.75) on a test set jointly established by two retinal experts, which consisted of 100 fundus images (10 poor-quality, 30 no MM, 30 TF and 30 PM) picked from the collected datasets. Secondly, all images were randomly divided among the four ophthalmologists for quality control checks so that unqualified images are excluded. Thirdly, each of the remaining gradable images was randomly assigned to any two of the four ophthalmologists for independent grading of MM severity levels (no MM, TF, and PM). Finally, those images with inconsistent third-tier grading results were randomly sent to one of the two retinal experts to determine the final outcomes. The grading results obtained in the above process were considered as the reference standard for this study; the distribution is shown in Table 1.

## Image processing

Fundus images captured from different cameras and sites vary in brightness, contrast, and color balance. To alleviate the influence caused by these variations, we developed an automated image processing method via color histogram distribution optimization (CHDO) based on the Age-Related Eye Disease Study 2 report [26]. It consists of the following steps: (1) selecting an optimal standardized image as the template image; (2) shifting the histogram distribution of color channels from the input image to the target image via a linear transformation that makes the average brightness and standard deviation values of blue, green, and red channels within the foreground region of the input image consistent with the template image; and (3) applying contrast limited adaptive histogram equalization [27] for enhancement.

## DCNN development

**Fig. 1:**
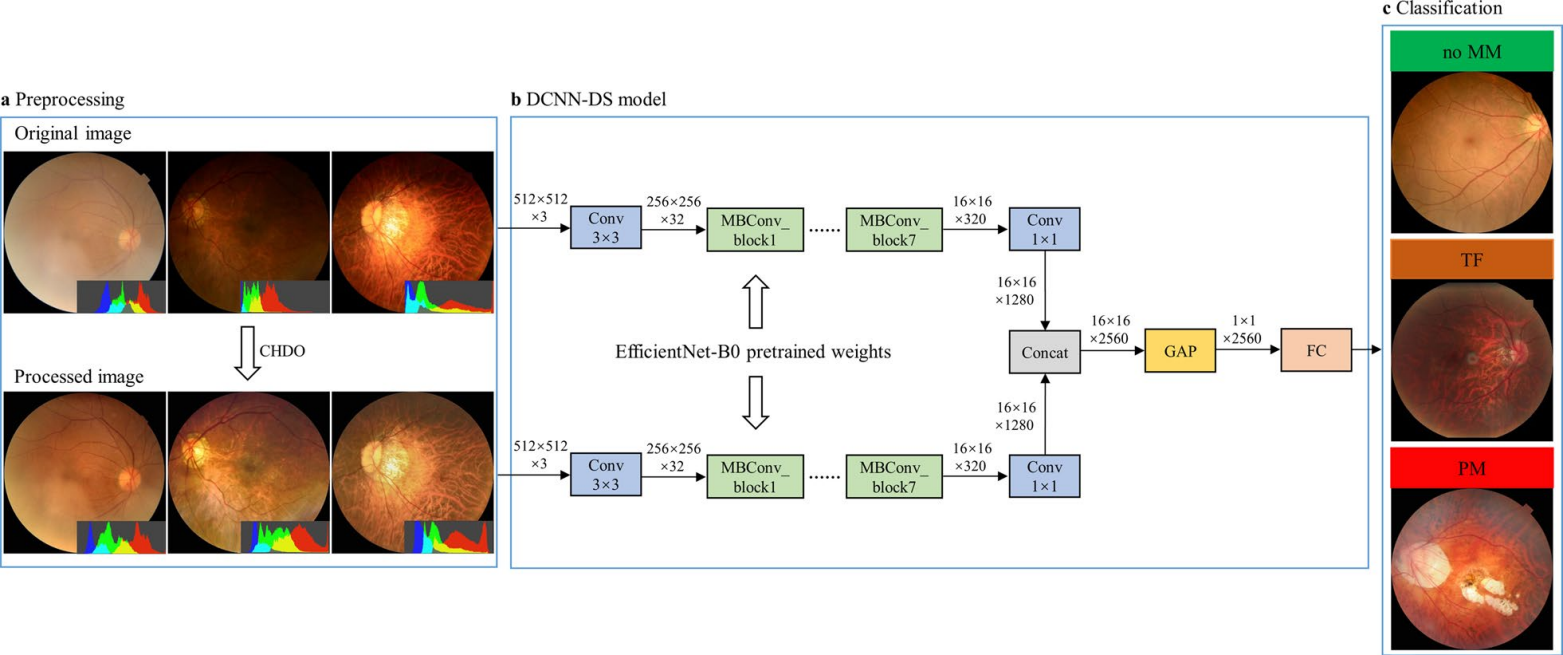
The framework of our proposed DCNN approach. **a** The processed images have more uniform color histogram distribution and better clarity than the original images in most cases. **b** Brief structure of the DCNN-DS model using both original and processed images as inputs. **c** The classification output into no MM, TF, or PM. MM, myopic maculopathy; TF, tessellated fundus; PM, pathologic myopia; CHDO, color histogram distribution optimization; Conv, convolution; MBConv, mobile inverted bottlrneck convolution; Concat, concatenation; GAP, global average pooling; FC, full connection

In this study, a dual-stream architecture-based DCNN (DCNN-DS) was used to classify different stages of MM, as shown in Fig. 1. The original image and corresponding image processed by the CHDO method were both cropped, padded, and resized to 512 × 512 pixel resolution then input to the upper and bottom branches, respectively. The two branches share the same network structure, using EfficientNet-B0 [28] as the backbone. The initial weights of EfficientNet-B0 were pretrained on the large public database of ImageNet [29]. Two 1280-channel feature maps separately generated from the upper and lower branches were concatenated as a 2560-channel feature map. The fused feature map was compressed to form a 2560-dim feature vector which was fed into a full connection layer then a SoftMax layer providing probability scores of no MM, TF, and PM. Besides, the predicted class score was mapped back to the fused feature map to generate the class activation map (CAM) [30] for highlighting the class-specific discriminative regions.

The DCNN-DS model combines informative features from the original and processed images. All gradable fundus images in the development dataset were randomly divided into two parts: 80% were used for training and the remaining 20% for validation. Focal loss [31] and Adam optimizer [32] were utilized for training on this multi-class classification task. The model with highest accuracy on the internal validation dataset was selected. For comparison, two single-stream EfficientNet-B0-based DCNNs from original images (DCNN-O) and processed images (DCNN-P) were built using the same datasets.

## Models testing

Two large datasets from the Shandong Eye Hospital (SDEH) and Qingdao Eye Hospital (QDEH) were used for external testing of the DCNN-DS model as well as the other two DCNN models. Details of SDEH and QDEH datasets are listed in Table 1. In addition, we constructed a comparison testing dataset by randomly sampling 3000 gradable fundus images from the two external testing datasets, of which 1488 and 1512 images were from SDEH and QDEH, respectively. Two junior testing ophthalmologists (LX and LXY) with less than two years of clinical experience and two senior testing ophthalmologists (QY and ZZF) with over five years of clinical experience were invited to independently label these images. The performances of four testing ophthalmologists and DCNN-DS model were assessed in this dataset according to the reference standard.

## Evaluation metrics

Three probability scores corresponding to no MM, TF, and PM were generated for each image passing through the DCNN models. The final classification result was determined by taking the category with the maximum probability score. Compared with the reference standard, we calculated the overall accuracies and κ scores of the DCNN models on the internal validation and external testing datasets. Sensitivity and specificity were assessed to measure the diagnostic accuracy of TF and PM according to the one-versus-rest strategy. In addition, we plotted the receiver operating characteristic curves of TF versus others (no MM + PM) and PM versus others (no MM + TF), then computed the AUCs to further assess diagnostic accuracy of different DCNN models to detect TF and PM. Two-tailed 95% confidence intervals (CIs) were also calculated for sensitivity, specificity, and AUC.

## Results

The development dataset included 40,594 fundus images obtained from four hospitals; 36,515 (89.9%) passed image quality control and were graded by ophthalmologists. Of these, 7302 images including 5292 (72.5%) no MM, 987 (13.5%) TF, and 1023 (14.0%) PM were randomly selected for internal validation. Table 2 shows the performances of the DCNN approaches both in four subsets broken down by image source and the overall validation dataset. Among the whole internal validation dataset, the overall accuracies of three models (DCNN-DS, DCNN-P, and DCNN-O) were 96.5%, 93.0%, and 90.5%, with κ scores of 0.922, 0.849, and 0.799, respectively. For detecting PM, the DCNN-DS model achieved a sensitivity of 96.4%, specificity of 99.2%, and an AUC of 0.997, followed by DCNN-P (sensitivity = 92.8%, specificity = 98.7%, AUC = 0.996), and DCNN-O (sensitivity = 88.8%, specificity = 97.8%, AUC = 0.991). For detecting TF, the DCNN-DS model achieved a sensitivity of 93.6%, specificity of 97.2%, and AUC of 0.985, followed by DCNN-P (sensitivity = 91.8%, specificity = 93.9%, AUC = 0.975), and DCNN-O (sensitivity = 91.2%, specificity = 91.9%, AUC = 0.966).

**Table 2:** Performance of the DCNN models on the internal validation and external testing datasets

Model	Accuracy (%)	κ	Sensitivity (%, 95% CI)		Specificity (%, 95% CI)		AUC (95% CI)	
			PM	TF	PM	TF	PM	TF
Internal validation								
Qingdao Eye Hospital North Branch of Shandong First Medical University (Camera: Canon, Syseye)								
DCNN-DS	96.5	0.922	96.6 (94.1–98.1)	94.7 (91.8–96.6)	99.3 (98.8–99.6)	97.0 (96.2–97.6)	0.998 (0.996–0.999)	0.989 (0.984–0.992)
DCNN-P	93.1	0.862	93.2 (90.1–95.5)	93.1 (90.0–95.3)	98.8 (98.2–99.1)	94.1 (93.1–95.0)	0.995 (0.992–0.997)	0.978 (0.972–0.983)
DCNN-O	90.5	0.800	88.8 (85.1–91.7)	91.9 (88.6–94.3)	97.9 (97.2–98.4)	91.8 (90.6–92.9)	0.991 (0.987–0.994)	0.968 (0.961–0.974)
Rongcheng Eye Hospital (Camera: Zeiss)								
DCNN-DS	96.8	0.928	97.8 (95.6–99.0)	91.4 (87.6–94.1)	99.0 (98.4–99.3)	97.7 (97.0–98.3)	0.998 (0.995–0.999)	0.983 (0.977–0.987)
DCNN-P	93.2	0.853	93.3 (90.1–95.5)	90.7 (86.9–93.6)	98.7 (98.0–99.1)	94.1 (93.0–95.0)	0.997 (0.994–0.999)	0.972 (0.965–0.978)
DCNN-O	90.3	0.796	90.8 (87.3–93.5)	89.2 (85.2–92.3)	97.6 (96.8–98.2)	92.2 (91.0–93.3)	0.992 (0.988–0.995)	0.962 (0.954–0.969)
Qilu Hospital of Shandong University (Qingdao) (Camera: Canon)								
DCNN-DS	95.6	0.903	93.8 (88.2–96.9)	92.6 (86.8–96.0)	99.3 (98.5–99.7)	96.4 (94.9–97.5)	0.997 (0.991–0.999)	0.984 (0.974–0.990)
DCNN-P	93.6	0.862	92.4 (86.5–96.0)	92.6 (86.8–96.0)	99.0 (98.0–99.5)	94.2 (92.4–95.6)	0.995 (0.989–0.998)	0.982 (0.972–0.988)
DCNN-O	90.4	0.798	83.4 (76.2–88.9)	93.2 (87.6–96.5)	98.3 (97.2–99.0)	91.4 (89.3–93.1)	0.989 (0.981–0.994)	0.966 (0.953–0.975)
No. 971 Hospital of the People’s Liberation Army (Camera: Topcon)								
DCNN-DS	96.9	0.928	94.3 (88.1–97.5)	97.5 (92.4–99.4)	99.3 (98.3–99.7)	97.3 (95.9–98.3)	0.989 (0.980–0.994)	0.981 (0.970–0.988)
DCNN-P	91.6	0.811	90.2 (83.1–94.6)	89.3 (82.1–94.0)	98.5 (97.4–99.2)	92.6 (90.5–94.3)	0.991 (0.983–0.995)	0.962 (0.948–0.972)
DCNN-O	91.0	0.802	88.5 (81.2–93.4)	91.8 (85.1–95.8)	97.9 (96.7–98.8)	92.0 (89.9–93.7)	0.988 (0.979–0.993)	0.968 (0.955–0.977)
Overall								
DCNN-DS	96.5	0.922	96.4 (95.0–97.4)	93.6 (91.9–95.0)	99.2 (98.9–99.4)	97.2 (96.8–97.6)	0.997 (0.995–0.998)	0.985 (0.982–0.988)
DCNN-P	93.0	0.849	92.8 (91.0–94.2)	91.8 (89.9–93.4)	98.7 (98.4–99.0)	93.9 (93.3–94.5)	0.996 (0.994–0.997)	0.975 (0.971–0.978)
DCNN-O	90.5	0.799	88.8 (86.6–90.6)	91.2 (89.2–92.8)	97.8 (97.5–98.2)	91.9 (91.2–92.6)	0.991 (0.989–0.993)	0.966 (0.962–0.970)
External testing								
Shandong Eye Hospital of Shandong First Medical University (Camera: Topcon)								
DCNN-DS	96.3	0.922	93.3 (90.6–95.2)	98.8 (98.1–99.2)	99.6 (99.5–99.8)	95.6 (95.0–96.1)	0.998 (0.997–0.999)	0.986 (0.983–0.988)
DCNN-P	93.9	0.872	88.1 (84.9–90.8)	92.5 (91.1–93.7)	98.7 (98.4–98.9)	94.5 (93.8–95.1)	0.995 (0.993–0.996)	0.972 (0.968–0.976)
DCNN-O	92.5	0.844	88.7 (85.6–91.3)	94.7 (93.5–95.7)	98.9 (98.7–99.2)	91.9 (91.1–92.6)	0.996 (0.994–0.997)	0.970 (0.966–0.974)
Qingdao Eye Hospital of Shandong First Medical University (Camera: Kowa)								
DCNN-DS	93.0	0.797	91.0 (88.5–93.0)	92.8 (90.6–94.5)	98.7 (98.4–98.9)	94.1 (93.5–94.6)	0.994 (0.992–0.995)	0.970 (0.966–0.974)
DCNN-P	92.3	0.772	79.8 (76.5–82.8)	87.1 (84.5–89.4)	98.5 (98.1–98.7)	93.8 (93.2–94.4)	0.990 (0.988–0.992)	0.967 (0.963–0.971)
DCNN-O	90.6	0.731	74.5 (70.9–77.7)	89.0 (86.5–91.1)	98.2 (97.9–98.5)	91.7 (91.1–92.4)	0.987 (0.984–0.989)	0.960 (0.955–0.964)

*AUC* = area under the curve; *CI* = confidence interval; *PM* = pathologic myopia; *TF* = tessellated fundus

**Fig. 2:**
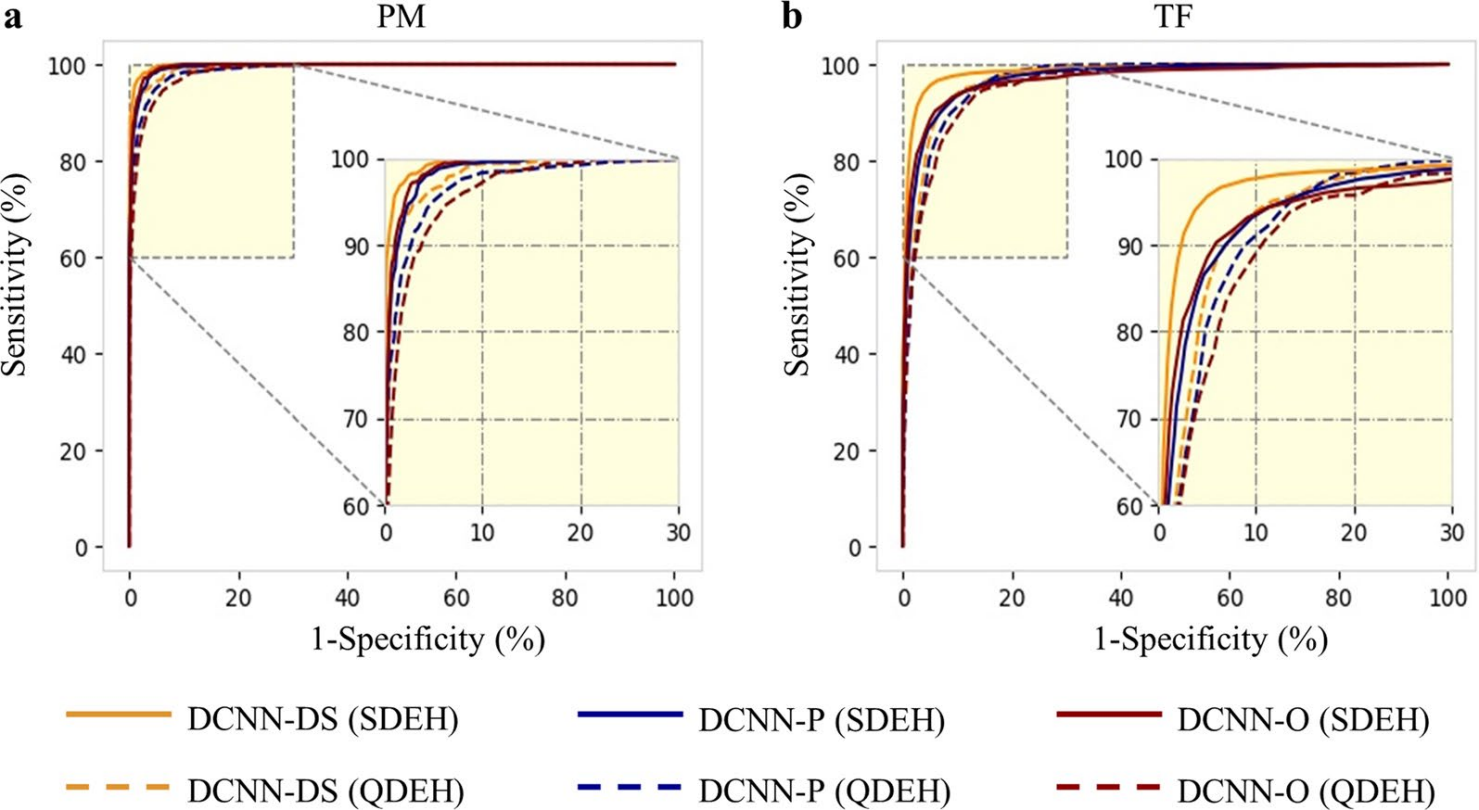
Receiver operating characteristic (ROC) curves of three DCNN models on the Shandong Eye Hospital (SDEH) and Qingdao Eye Hospital (QDEH) external testing datasets. **a** ROC curves for PM; **b** ROC curves for TF. DCNN, deep convolutional neural network; PM, pathologic myopia; TF, tessellated fundus

**Fig. 3:**
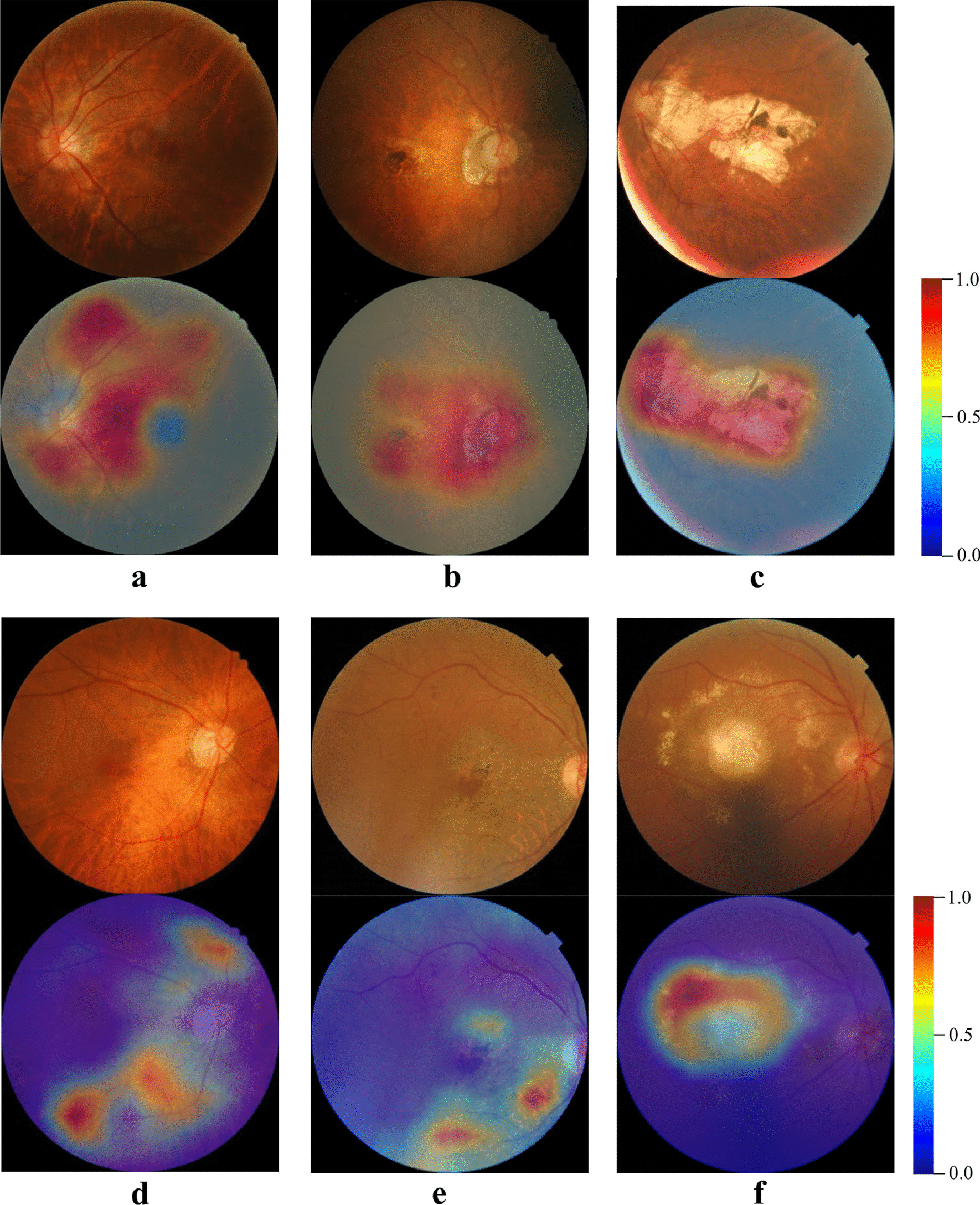
Typical examples of true positive, false negative and false positive images and the corresponding CAM heatmaps. **a** Is a true positive of TF, the heatmap predominantly visualizes TF region. **b**, **c** Are true positives of PM respectively, the corresponding heatmaps highlight the atrophy lesions. In the row below, **d** is a false negative of PM which is recognized as TF by the DCNN-DS model, **e**, **f** are false positives of TF and PM respectively caused by other macular pathologies. The corresponding heatmaps also visualize the major interested regions of the DCNN-DS model. CAM, class activation map; TF, tessellated fundus; PM, pathologic myopia; DCNN-DS, dual-stream deep convolutional neural network

The SDEH and QDEH datasets, which were used for external testing of the DCNN models, consisted of 7077 [506 (7.2%) PM, 1687 (23.8%) TF] and 7909 [654 (8.3%) PM, 747 (9.4%) TF] gradable images, respectively. The performances of three DCNN models for these two datasets are presented in Table 2 and Fig. 2. In the SDEH dataset, the accuracies were 96.3%, 93.9%, and 92.5%, the κ scores were 0.922, 0.872, and 0.844 for the DCNN-DS, DCNN-P and DCNN-O models, respectively. For detecting PM, the DCNN-DS model achieved a sensitivity of 93.3%, specificity of 99.6%, and AUC of 0.998, followed by DCNN-P (sensitivity = 88.1%, specificity = 98.7%, AUC = 0.995), and DCNN-O (sensitivity = 88.7%, specificity = 98.9%, AUC = 0.996). For detecting TF, the DCNN-DS model achieved a sensitivity of 98.8%, specificity of 95.6%, and AUC of 0.986, followed by DCNN-P (sensitivity = 92.5%, specificity = 94.5%, AUC = 0.972), and DCNN-O (sensitivity = 94.7%, specificity = 91.9%, AUC = 0.970). In the QDEH dataset, the accuracies were 93.0%, 92.3%, and 90.6%, and the κ scores were 0.797, 0.772, and 0.731 for the DCNN-DS, DCNN-P and DCNN-O models, respectively. For detecting PM, the DCNN-DS model achieved a sensitivity of 91.0%, specificity of 98.7%, and AUC of 0.994, followed by DCNN-P (sensitivity = 79.8%, specificity = 98.5%, AUC = 0.990), and DCNN-O (sensitivity = 74.5%, specificity = 98.2%, AUC = 0.987). For detecting TF, the DCNN-DS model achieved a sensitivity of 92.8%, specificity of 94.1%, and AUC of 0.970, followed by DCNN-P (sensitivity = 87.1%, specificity = 93.8%, AUC = 0.967), and DCNN-O (sensitivity = 89.0%, specificity = 91.7%, AUC = 0.960). Figure 3 shows some typical examples of true positive as well as false negative and false positive images recognized by the DCNN-DS model, together with the CAMs that have been superimposed on the images.

**Fig. 4:**
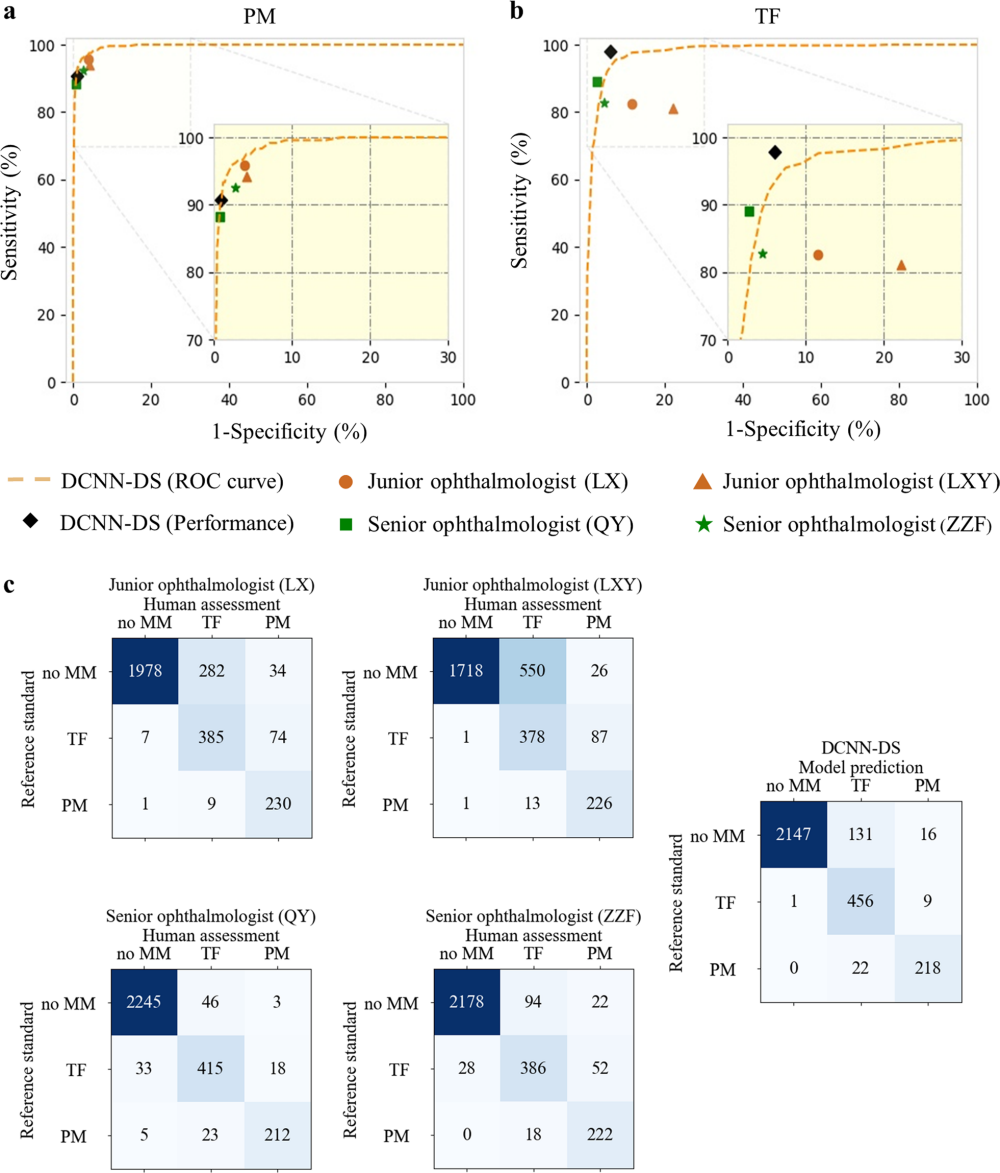
Results for the comparison testing dataset comparing our DCNN model with four ophthalmologists. **a**, **b** Performance of the DCNN-DS model and ophthalmologists for the detection of PM and TF, respectively. **c** Five confusion matrices for our DCNN model and ophthalmologists; the numbers of correct classification are listed on the diagonal. DCNN, deep convolutional neural network; PM, pathologic myopia; TF, tessellated fundus; DCNN-DS, dual-stream DCNN

The comparison results of the DCNN-DS model and four testing ophthalmologists of different tiers on the sampled testing dataset of 3000 [240 (8.0%) PM, 466 (15.5%) TF] images are shown in Fig. 4. The overall accuracies and κ scores of the ophthalmologists ranged from 77.4% to 95.7% and 0.551 to 0.890, while the DCNN-DS model achieved 94.0% accuracy with a κ score of 0.856. For detecting PM, ophthalmologist sensitivities ranged from 88.3% to 95.8%, and the specificities ranged from 95.9% to 99.2%. The average sensitivity and specificity of two junior ophthalmologists were 95.0% and 96.0%, while those of two senior ophthalmologists were 90.4% and 98.3%, respectively. Meanwhile, the sensitivity, specificity, and AUC of the DCNN-DS model were 90.8%, 99.1%, and 0.996, respectively. For detecting TF, ophthalmologist sensitivities ranged from 81.1% to 89.1%, and the specificities ranged from 77.8% to 97.3%. The average sensitivity and specificity of two junior ophthalmologists were 81.9% and 83.2%, while those of two senior ophthalmologists were 85.9% and 96.5%, respectively. The sensitivity, specificity, and AUC of the DCNN-DS model were 97.9%, 94.0%, and 0.979, respectively.

## Discussion

In this study, we aimed to develop DCNN-based methods for the automated detection of MM with high reliability from color fundus images. The major highlights of our study lies in three aspects: (1) building up a large database of color fundus photographs from six medical centers and performing a consistent multi-tier grading process for the MM research; (2) addressing a novel image preprocessing methodology to prepare color fundus images for use in a dual-steam DCNN model; and (3) thoroughly validating the proposed DCNN model using two large external testing datasets and executing comparative experiments with ophthalmologists to demonstrate the reliability of our proposed DCNN approach.

Several studies achieved promising results on the lesions segmentation and binary classification of PM by applying DCNN approaches on a public dataset containing 1200 fundus images. More extensive validations were required to demonstrate generalizability of these DCNN models [21, 22]. Tan et al. [23] collected 226,686 fundus images from nine multiethnic cohorts from six regions and performed thorough validations of the developed deep learning algorithms for classifying MM and HM. The scale and diversity of the datasets used in this study were quite impressive, while the annotations of MM and HM were not conducted by uniform graders and were not complete in the external testing datasets. Among three external testing datasets, the reported sensitivities of detecting MM were higher than 94.0%, whereas the specificities varied unstably from 85.5% to 95.9%. As for HM, the model performance demonstrated larger gaps in those independent external testing datasets. Du et al. [24] conducted a single-center study that recruited 7020 fundus images to develop and validate a DCNN algorithm to detect PM and to categorize the different MM lesions. This study attained an accuracy of 92.1% in the internal validation dataset in differentiating non-PM from PM, whereas the accuracies in two external testing datasets were 78.1% and 88.2%, respectively. Lu et al. [25] developed DCNN models to classify the categories of MM and further detect the plus lesions using a dataset of 32,010 fundus images collected from three medical centers. By contrast, the scale of external testing dataset which contained 1000 fundus images adopted in the study was insufficient.

At the beginning, a total of 57,148 color fundus images from 29,230 patients captured by a range of fundus cameras in six hospitals were collected in this work. To ensure the accuracy and consistency of data labeling, all the fundus images were subjected to strict grading processes including image quality control, independent grading, and disagreement adjudication, which were conducted by four ophthalmologists and two specialists from QDEH. Rather than making binary classification between MM and no MM, this study further distinguished TF and PM from fundus photographs based on the META-PM criterion. This classification can help patients and ophthalmologists identify the progression stage of MM [3]. TF in those well-defined choroidal vessels can be observed clearly around the fovea and the arcade vessels is generally agreed to be the earliest clinical sign of MM. Meanwhile, PM with sight-threatening pathological changes such as diffuse chorioretinal atrophy, patchy chorioretinal atrophy, macular atrophy and other plus lesions needs further diagnosis or treatment in retinal clinics.

A recent survey [33] reported that many image-related factors had impacts on the performance of the deep learning algorithms for diabetic retinopathy, such as imaging conditions, dataset sources and scales, etc. Here, these factors were also taken into consideration for the intelligent analysis of MM. The fundus images used in this study possessed high diversity due to different environmental conditions and camera devices. Hence, we developed a novel image preprocessing method (CHDO) to increase uniformity of the color histogram distribution and to enhance contrast of the fundus images, but also might bring different degrees of color distortion in the processed images compared with the original ones. Both the original and processed images were complementary to each other and could provide useful features for the identification of MM. Consequently, we adopted a dual-stream structure based DCNN approach to extract features from original images and processed images separately and then make the final predictions based on the fused feature maps. In this way, it supplied a stacking effect of two single-stream models and balanced the contribution of the two branches dynamically during the training stage. To validate the effectiveness of the proposed DCNN-DS model, the DCNN-O and DCNN-P models that used the original and optimized images as input were built for comparison.

Two independent large-scale external testing datasets from SDEH and QDEH were used to evaluate DCNN model performance. For both datasets, the DCNN-DS model achieved the best performance with respect to overall accuracy and κ score, as well as the sensitivity, specificity, and AUC for detecting PM and TF. The DCNN-P model performed better than the DCNN-O model on most metrics. Collectively, the results demonstrate that applying the proposed preprocessing method can significantly improve performance by reducing deviations among multi-source datasets. The DCNN-DS model learning the fused features of the original and optimized images can further promote the performance and achieved sensitivities of more than 91.0% and 92.8%, specificities of more than 98.7% and 94.1%, and AUCs of more than 0.994 and 0.970 for identifying PM and TF across two independent external datasets, respectively. Especially in the QDEH dataset, where the fundus images were captured by the Kowa camera that did not appear in the development dataset, we observed that the DCNN-DS model was also able to improve the reliability for identifying PM distinctly. The sensitivity of PM was 74.5% by DCNN-O model compared with 91.0% by DCNN-DS model, the AUC of PM was 0.987 by DCNN-O model compared with 0.994 by DCNN-DS model. The inclusion of CAM visualization that highlights the possible pathological regions in positive cases would aid in understanding the decision process of the proposed DCNN-DS model to some degree.

We also compared the performance of the DCNN-DS model with four testing ophthalmologists on a subset of 3000 fundus images randomly sampled from the two external testing datasets. These four ophthalmologists with varying expertise showed inconsistent understanding of MM and consequently reported different results in the comparison testing dataset. The ophthalmologists performed comparably in terms of PM, which is clinically important. However, distinct differences in recognizing TF were found between the junior and senior ophthalmologists. In contrast, the proposed DCNN-DS model demonstrated close to ophthalmologist-level ability to detect PM and performed more reliably for TF.

Several limitations of this investigation should be considered. First, this study simplified the five categories of MM as described in META-PM into three and was unable to identify specific lesions, which would warrant further research. Second, the adopted MM grading criteria mainly focused on macular pathologies and disregarded other myopic findings that occurred in the peripheral retina and optic nerve. In addition to the basic fundus photograph examination, more advanced tests such as optical coherence tomography and wide-field fundus imaging are needed [34]. Third, we conducted a quality control check to exclude poor-quality images. However, it is inevitable that such quality issues will be encountered on fundus images captured in real-world settings. Automatic image quality evaluation [35, 36] is necessary to identify unqualified images and bring these to the attention of operators. Fourth, all the fundus images were collected in ophthalmic clinics. Other realistic settings such as community disease-screening and health examination centers were not covered by this study. The population characteristics and prevalence of MM in these settings are quite different from those of ophthalmic clinics, and these factors may affect DCNN model performance.

## Conclusions

In summary, the results of this study reveal that our proposed DCNN model can achieve robust performance for detecting TF and PM amongst numerous fundus images from different imaging settings. This approach shows great potential for automated identification of MM at different severity levels in high-risk populations. Further research should focus on evaluating the feasibility and cost-effectiveness of applying our DCNN model in real-world applications.

## Data Availability

The development and external testing datasets that we used in this study are sourced from six hospitals [Qingdao Eye Hospital of Shandong First Medical University, Qingdao Eye Hospital North Branch of Shandong First Medical University, Shandong Eye Hospital of Shandong First Medical University, Rongcheng Eye Hospital, 971 Hospital of PLA Navy and Qilu Hospital of Shandong University (Qingdao)]. The availability of these datasets is restricted under the agreement for the current study, so we are unable to make the datasets publicly available immediately. However, datasets and corresponding annotations can be made available upon reasonable request and with the permission of the respective research institutes. Please contact the first author Jun Li (doctor_li@126.com) for the request of data access.

## Abbreviations

HM: High myopia
PM: Pathologic myopia
MM: Myopic maculopathy
DCNN: Deep convolutional neural network
AUC: Area under the receiver operating characteristic curve
TF: Tessellated fundus
CHDO: Color histogram distribution optimization
DCNN-DS: Dual-stream deep convolutional neural network
CI: Confidence interval
